# Characteristics and Outcomes of Geriatric Patients With Orthopedic Injuries Presenting to the Emergency Department

**DOI:** 10.7759/cureus.108138

**Published:** 2026-05-02

**Authors:** Wareesha Bint E Fayyaz, Haider Ali, Junaid Shafiq, Muhammad Ziad, Shah Hussain, Waqar Ahmad, Mavish Arif

**Affiliations:** 1 Geriatric Medicine, Countess of Chester Hospital, Chester, GBR; 2 Orthopedic Surgery, Shaikh Zayed Postgraduate Medical Institute, Lahore, PAK; 3 Orthopedic Surgery, Dr. Abdul Samad Memorial Hospital, Thinadhoo, MDV; 4 Orthopedics, Mayo Hospital, Lahore, PAK; 5 Emergency, Lady Reading Hospital, Peshawar, PAK; 6 Orthopedics, District Headquarters (DHQ) Teaching Hospital, Medical Teaching Institution (MTI), Bannu, PAK; 7 Internal Medicine, Peshawar General Hospital, Peshawar, PAK

**Keywords:** comorbidities, complications, geriatric trauma, mortality, orthopedic injuries

## Abstract

Background: Geriatric trauma is an increasingly important public health concern, with orthopedic injuries representing a major cause of emergency department visits and hospitalizations among older adults.

Objective: The primary objective of this study was to assess the characteristics, injury patterns, emergency department presentations, and clinical outcomes of geriatric patients with orthopedic injuries.

Materials and methods: This descriptive cross-sectional study was conducted in the Department of Orthopedics at Allama Iqbal Teaching Hospital, Dera Ghazi Khan, from April 2025 to October 2025. A total of 325 patients aged ≥60 years presenting with radiologically confirmed orthopedic injuries were included using non-probability consecutive sampling. Data regarding demographics, comorbidities, mechanisms of injury, emergency presentation, management, complications, and outcomes were collected.

Results: The mean age of patients was 71.8 ± 7.4 years, and females accounted for 56.6% of the cohort. Comorbidities were present in 73.2% of patients, most commonly hypertension (52.9%) and diabetes mellitus (39.1%). Falls were the leading mechanism of injury (67.4%), followed by road traffic accidents (19.4%). Fractures were identified in 80.6% of cases, with hip fractures being the most common (37.2%). On arrival, 29.8% of patients were hemodynamically unstable, and 16% had a Glasgow Coma Scale score <13. A total of 204 patients (62.8%) required hospital admission, and 42.8% underwent operative management. Complications occurred in 21.8% of cases, including pneumonia (5.5%), delirium (4.9%), pressure ulcers (3.7%), and deep vein thrombosis (2.8%). The in-hospital mortality rate was 5.2%.

Conclusion: Geriatric patients presenting with orthopedic injuries demonstrate a high burden of comorbidities, predominantly fall-related trauma, and fracture patterns consistent with age-related fragility.

## Introduction

Orthopedic injuries in the geriatric population are rapidly emerging as a critical public health concern, particularly as global demographics shift toward an aging society [1]. The number of individuals older than 60 years is predicted to surpass 2.1 billion, greatly increasing the worldwide burden of age-related injuries [2]. Aging is associated with numerous changes, including decreases in bone mineral density, proprioception, and reaction time, as well as increased frailty and multimorbidity, all of which heighten the risk of trauma-related injuries [3]. Consequently, older adults are more likely to experience fractures, dislocations, soft tissue injuries, and trauma-related complications, even from relatively minor incidents.

Falls are a major contributor to injury in this population, accounting for approximately 60% of emergency department visits for injuries among older adults. They also contribute substantially to disability, hospitalization, and mortality in this age group [4]. A variety of factors influence outcomes in older patients with fractures, both non-modifiable (systemic) and modifiable. Low-energy mechanisms, such as ground-level falls, commonly result in fractures of the hip, vertebrae, and wrist [5]. These injuries can be particularly severe due to underlying osteoporosis and other degenerative conditions. Although less common, high-energy trauma can also lead to serious complications, often exacerbated by reduced physiological reserves [6]. Surgical complications in this population are likewise more frequent and severe [7].

Orthopedic injuries in older adults represent a substantial global public health burden. The World Health Organization estimates that 28-35% of adults aged 65 and older experience at least one fall annually, rising to 32-42% among those aged 70 and older. Falls are responsible for nearly 684,000 deaths worldwide each year. A recent meta-analysis reported a global prevalence of falls in older adults of 26.5% (95% confidence interval: 23.4-29.8), with significant regional variation. Furthermore, more than 50% of injury-related hospitalizations among adults over 65 are attributed to falls, underscoring their major contribution to morbidity, disability, and healthcare utilization [8,9].

These injuries also impose significant psychosocial and economic burdens on patients, families, and healthcare systems. Geriatric orthopedic trauma is associated with high rates of morbidity and mortality, often resulting in loss of functional independence, reduced mobility, and diminished ability to live independently. In-hospital mortality rates in geriatric trauma populations range from 15% to 45%, reflecting the complex, multifactorial nature of these injuries. Among individuals over 65, falls and trauma have become the fifth leading cause of death [10].

This vulnerability is further compounded by age-related physiological decline, even as many older adults maintain higher levels of physical activity compared to previous generations [11]. While fatal injuries are a major concern, even relatively minor injuries can lead to significant morbidity and mortality in older patients, making their overall impact particularly substantial [12].

The primary objective of this study is to determine the characteristics, injury patterns, and clinical outcomes of geriatric patients presenting to the emergency department with orthopedic injuries. The secondary objectives are to assess factors associated with in-hospital complications among geriatric orthopedic trauma patients and to evaluate factors associated with in-hospital mortality, including injury characteristics, hemodynamic instability, and neurological status at presentation.

## Materials and methods

This descriptive cross-sectional study was conducted at the Department of Orthopedics, Allama Iqbal Teaching Hospital, Dera Ghazi Khan, from April to October 2025. The Institutional Review Board of Allama Iqbal Teaching Hospital issued approval AITH/IRB/2025/231. A total of 325 geriatric patients were included. The sample size was calculated using the WHO sample size calculator for estimating a single population proportion. Assuming an expected frequency of orthopedic injuries among geriatric trauma patients of 30%, with a 95% confidence level and a 5% margin of error, the minimum required sample size was 323 patients, rounded up to 325.

Patients aged 60 years or older, of either gender, presenting to the emergency department with radiologically confirmed orthopedic injuries were included. Eligible cases comprised fractures, dislocations, or orthopedic trauma involving the upper limb, lower limb, pelvis, or spine. Both early and delayed presentations were included, provided that complete clinical, radiological, management, and outcome data were available. Patients with isolated soft tissue injuries without radiological evidence of orthopedic involvement were excluded. Those who were dead on arrival, had incomplete medical records, lacked radiological confirmation, or had unavailable outcome data were also excluded. Additionally, patients referred after definitive orthopedic management at another facility were excluded to avoid duplication or misclassification of initial emergency care outcomes.

Data collection

Data were recorded using a structured proforma by trained emergency department staff. Variables included demographic characteristics (age and gender), comorbidities (hypertension, diabetes, cardiovascular disease, and osteoporosis), mechanism of injury (fall, road traffic accident, assault, or others), type and anatomical site of injury, vital signs on arrival, and Glasgow Coma Scale (GCS) scores, where available. Radiological diagnosis was performed using X-rays, CT scans, or MRI, based on clinical judgment. Initial management, including immobilization, analgesia, splinting, and referral for operative or non-operative treatment, was also recorded.

Radiological diagnosis followed a standardized imaging approach based on injury type. Plain X-rays were used as the primary imaging modality for all suspected orthopedic injuries. At the same time, CT scans or MRI were utilized when clinically indicated for complex, occult, spinal, or intra-articular injuries. Consultant radiologists and orthopedic specialists conducted imaging interpretation in accordance with routine institutional diagnostic protocols to minimize diagnostic variability and classification bias.

Short-term outcomes, including the need for hospital admission, length of stay, procedures performed, associated complications, and in-hospital mortality, were also documented. Missing data were minimized by including only patients with complete clinical, radiological, management, and outcome records. Cases with missing key variables relevant to outcome analysis were excluded using complete-case analysis. As the proportion of missing data was minimal, no imputation methods were applied. This approach was adopted to reduce information bias and maintain consistency in statistical comparisons.

Data analysis

Data were entered and analyzed using SPSS Statistics version 26.0 (IBM Corp. Released 2019. IBM SPSS Statistics for Windows, Version 26.0. Armonk, NY: IBM Corp.). Quantitative variables, such as age and length of hospital stay, were presented as mean ± standard deviation. Categorical variables, including gender, mechanism of injury, comorbidities, and outcomes, were expressed as frequencies and percentages. The chi-square test was used to assess associations between injury characteristics and outcomes. A p-value ≤ 0.05 was considered statistically significant.

## Results

Data were collected from 325 patients. The mean age of participants was 71.8 ± 7.4 years, with the largest proportion, 138 (42.5%), aged 60-69 years. Females constituted 184 (56.6%) of the cohort.

A substantial comorbidity burden was observed, with 238 (73.2%) patients having at least one chronic medical condition. The most common comorbidities were hypertension in 172 (52.9%), diabetes mellitus in 127 (39.1%), and osteoporosis in 89 (27.4%) patients.

Falls were the predominant mechanism of injury, accounting for 219 (67.4%) cases, followed by road traffic accidents (63, 19.4%) and other low-energy trauma mechanisms (28, 8.6%). Fractures accounted for 80.6% of all injuries. Among fracture sites, hip fractures were most frequent (121, 37.2%), followed by wrist/forearm fractures (68, 20.9%) and vertebral fractures (44, 13.5%). Polytrauma was observed in 18 patients (5.5%) (Table 1).

**Table 1:** Sociodemographic characteristics of geriatric patients (n = 325) SD: standard deviation

Variable	Category	n (%)/mean ± SD
Age (years)	Mean ± SD	71.8 ± 7.4
Age group	60-69 years	138 (42.5)
	70-79 years	116 (35.7)
	≥80 years	71 (21.8)
Gender	Male	141 (43.4)
	Female	184 (56.6)
Comorbidities (overall)	Present	238 (73.2)
	Absent	87 (26.8)
Individual comorbidities	Hypertension	172 (52.9)
	Diabetes mellitus	127 (39.1)
	Osteoporosis	89 (27.4)
	Ischemic heart disease	54 (16.6)
	Chronic kidney disease	37 (11.4)
Mechanism of injury	Falls (ground-level/same-level)	219 (67.4)
	Road-traffic accident	63 (19.4)
	Non-fall low-impact trauma*	28 (8.6)
	Other (assault/heavy object injury)	15 (4.6)
Type of injury	Fracture	262 (80.6)
	Dislocation	41 (12.6)
	Soft-tissue injury	22 (6.8)
Fracture site	Hip	121 (37.2)
	Wrist/forearm	68 (20.9)
	Vertebrae	44 (13.5)
	Shoulder/proximal humerus	29 (8.9)
	Pelvis	14 (4.3)
Polytrauma	Present	18 (5.5)

Most patients (228, 70.2%) arrived hemodynamically stable, while 97 (29.8%) were unstable at presentation. The majority (273, 84.0%) had a GCS score ≥13, whereas 52 (16.0%) presented with reduced level of consciousness. Pain intensity was predominantly moderate to severe in more than 87% of cases.

A total of 204 patients (62.8%) required hospital admission, and 139 (42.8%) underwent operative management. The mean length of hospital stay was 6.4 ± 3.1 days. Complications occurred in 21.8% of patients, with pneumonia, delirium, pressure ulcers, deep vein thrombosis, and wound infections being the most frequently reported (Table 2).

**Table 2:** Emergency presentation and initial assessment (n = 325) GCS: Glasgow Coma Scale

Variable	Category	n (%)
Hemodynamic stability	Stable	228 (70.2%)
	Unstable	97 (29.8%)
GCS on arrival [13]	≥13	273 (84.0%)
	<13	52 (16.0%)
Pain severity	Mild	42 (12.9%)
	Moderate	128 (39.4%)
	Severe	155 (47.7%)
Hospital admission	Admitted	204 (62.8%)
	Discharged from ED	121 (37.2%)
Type of management	Operative	139 (42.8%)
	Non-operative	186 (57.2%)
Length of hospital stay (days)	Mean ± SD	6.4 ± 3.1
Common complications	Pneumonia	18 (5.5%)
	Delirium	16 (4.9%)
	Pressure ulcers	12 (3.7%)
	Deep vein thrombosis	9 (2.8%)
	Wound infection	11 (3.4%)
Overall complications	Any complication	71 (21.8%)
Mortality	In-hospital deaths	17 (5.2%)

Increasing age was significantly associated with higher complication rates, with patients aged ≥80 years having the highest complication rate (40.8%) compared with younger age groups (p = 0.002). Osteoporosis was also strongly associated with complications (40.8% vs. 23.6%, p = 0.004). Hemodynamic instability at presentation emerged as a highly significant predictor of adverse outcomes, with 46.5% of unstable patients experiencing complications compared with 25.2% of stable patients (p < 0.001) (Table 3).

**Table 3:** Association between patient characteristics and development of complications (n = 325)

Variable	Category	Complications present (n = 71)	Complications absent (n = 254)	Test statistic (χ²)	p-value
Age group	60-69 years	18 (25.4%)	120 (47.2%)	12.41	0.002
	70-79 years	24 (33.8%)	92 (36.2%)		
	≥80 years	29 (40.8%)	42 (16.5%)		
Gender	Male	29 (40.8%)	112 (44.1%)	0.29	0.59
	Female	42 (59.2%)	142 (55.9%)		
Comorbidities (overall)	Present	58 (81.7%)	180 (70.9%)	3.52	0.06
	Absent	13 (18.3%)	74 (29.1%)		
Osteoporosis	Present	29 (40.8%)	60 (23.6%)	8.32	0.004
	Absent	42 (59.2%)	194 (76.4%)		
Hemodynamic instability	Present	33 (46.5%)	64 (25.2%)	12.92	<0.001
	Absent	38 (53.5%)	190 (74.8%)		

Although hip fractures showed a significant univariate association with in-hospital mortality (64.7% vs. 35.7%, p = 0.013), polytrauma demonstrated the strongest association, with a mortality rate of 35.3% in this subgroup compared with 3.9% among survivors (p < 0.001). Low GCS scores (<13) and hemodynamic instability were also strong predictors of mortality, both showing highly significant associations (p < 0.001) (Table 4).

**Table 4:** Association between injury characteristics and in-hospital mortality (n = 325) GCS: Glasgow Coma Scale

Variable	Category	Mortality present n (%)	Mortality absent n (%)	Adjusted OR (95% CI)	p-value
Hip fracture	Yes	11 (64.7)	110 (35.7)	1.84 (0.76-4.43)	0.17
	No	6 (35.3)	198 (64.3)	Reference	-
Polytrauma	Yes	6 (35.3)	12 (3.9)	5.92 (1.94-18.07)	0.002
	No	11 (64.7)	296 (96.1)	Reference	-
GCS <13	Yes	11 (64.7)	41 (13.3)	6.75 (2.24-20.38)	<0.001
	No	6 (35.3)	267 (86.7)	Reference	-
Hemodynamic instability	Yes	12 (70.6)	85 (27.6)	4.88 (1.61-14.76)	0.005
	No	5 (29.4)	223 (72.4)	Reference	-
Age ≥80 years	Yes	8 (47.1)	63 (20.5)	2.21 (0.88-5.52)	0.09
	No	9 (52.9)	245 (79.5)	Reference	-

## Discussion

This study explored the demographic patterns, injury characteristics, emergency department presentations, and clinical outcomes of geriatric patients presenting with orthopedic injuries. With 325 patients included, the findings highlight the growing burden of geriatric trauma and its clinical as well as system-level implications. The mean age of the study population was 71.8 years, with nearly one-fifth aged 80 years and older. This age distribution is consistent with global trends, reflecting increased life expectancy and a growing elderly population.

Females constituted 56.6% of the cohort, likely reflecting their longer life expectancy and the higher prevalence of osteoporosis and consequent fragility fractures in this group [14]. The substantial comorbidity burden, particularly hypertension (52.9%), diabetes mellitus (39.1%), and osteoporosis (27.4%), is consistent with previous studies showing that most geriatric trauma patients present with multiple chronic conditions that complicate both assessment and management.

Falls accounted for more than two-thirds of all injuries and remain the most common mechanism of injury in older adults, as widely reported in the literature [15]. Contributing factors include impaired balance, slower gait, reduced visual acuity, and diminished neuromuscular and cognitive function. The risk is further increased by polypharmacy, particularly the use of sedatives, antihypertensives, and psychotropic medications [16]. Although less frequent, road traffic accidents (19.4%) were associated with more severe injury patterns, consistent with evidence that older adults sustain significant trauma even in low-impact events due to reduced bone density and slowed reflexes.

The observed injury patterns are also consistent with established literature. Hip fractures were the most common injury (37.2%), aligning with global data identifying them as the most clinically significant fractures in older adults due to osteoporosis and frailty, particularly following low-energy falls [17]. Wrist and forearm fractures (20.9%) and vertebral fractures (13.5%) were also frequently observed, reflecting typical fall-related injury distributions in this population. Polytrauma was less common (5.5%) but was predominantly associated with road traffic accidents and carried greater severity.

A total of 62.8% of patients required hospital admission, and 42.8% underwent operative management. These findings are consistent with existing literature indicating that hip and long-bone fractures in geriatric patients often require surgical stabilization to reduce complications such as thromboembolism, pressure ulcers, and pneumonia. However, decisions regarding surgery are often influenced by frailty, comorbidities, and anesthetic risk. The mean hospital stay of 6.4 days is comparable to international reports but still represents a substantial utilization of healthcare resources in tertiary care settings [18].

Complications occurred in 21.8% of patients, with pneumonia, delirium, pressure ulcers, deep vein thrombosis, and wound infections being the most common. These complications are well-recognized consequences of immobility, physiological stress, and polypharmacy in older trauma patients. Notably, pneumonia and delirium are associated with prolonged hospitalization and increased mortality [19]. These findings support the need for early mobilization protocols, vigilant perioperative monitoring, and multidisciplinary geriatric involvement in trauma care.

The study also highlights systemic challenges in geriatric emergency care, particularly in low-resource settings, where limited pre-hospital services, delayed presentation, and restricted access to imaging and structured geriatric trauma pathways may worsen outcomes. The findings, therefore, reflect both clinical patterns and health system constraints relevant to the South Asian context [20].

However, the study has limitations. Its cross-sectional design precludes causal inference, and the single-center setting may limit generalizability to rural or peripheral healthcare facilities. Additionally, long-term functional outcomes and quality-of-life measures were not assessed. Despite these limitations, the study provides a useful foundation for future research, which may benefit from incorporating frailty scoring systems, long-term follow-up, rehabilitation outcomes, and comparative study designs to improve prognostic accuracy and clinical decision-making.

## Conclusions

Geriatric patients presenting with orthopedic injuries constitute a clinically vulnerable population characterized by a high burden of comorbidities, predominantly fall-related trauma, and injury patterns strongly associated with age-related fragility, particularly hip, wrist, and vertebral fractures. A significant proportion required hospital admission and surgical intervention, while more than one-fifth developed in-hospital complications, reflecting reduced physiological reserve and the complexity of managing trauma in older adults.
